# Protective Role of Intracellular Melatonin Against Oxidative Stress and UV Radiation in *Saccharomyces cerevisiae*

**DOI:** 10.3389/fmicb.2018.00318

**Published:** 2018-02-28

**Authors:** Ricardo Bisquert, Sara Muñiz-Calvo, José M. Guillamón

**Affiliations:** Departamento de Biotecnología de Alimentos, Instituto de Agroquímica y Tecnología de Alimentos, Consejo Superior de Investigaciones Científicas (CSIC), Valencia, Spain

**Keywords:** melatonin, *Saccharomyces cerevisiae*, UV radiation, oxidative stress, gene expression

## Abstract

Melatonin (Mel) is considered a potent natural antioxidant molecule given its free-radical scavenging ability. Its origin is traced back to the origin of aerobic life as early defense against oxidative stress and radiation. More complex signaling functions have been attributed to Mel as a result of evolution in different biological kingdoms, which comprise gene expression modulation, enzyme activity, and mitochondrial homeostasis regulation processes, among others. Since Mel production has been recently reported in wine yeast, we tested the protective effect of Mel on *Saccharomyces cerevisiae* against oxidative stress and UV light. As the optimal conditions for *S. cerevisiae* to synthesize Mel are still unknown, we developed an intracellular Mel-charging method to test its effect against stresses. To assess Mel’s ability to protect *S. cerevisiae* from both stresses, we ran growth tests in liquid media and viability assays by colony count after Mel treatment, followed by stress. We also analyzed gene expression by qPCR on a selection of genes involved in stress protection in response to Mel treatment under oxidative stress and UV radiation. The viability in the Mel-treated cells after H_2_O_2_ stress was up to 35% greater than for the untreated controls, while stress amelioration reached 40% for UVC light (254 nm). Mel-treated cells showed a significant shortened lag phase compared to the control cells under the stress and normal growth conditions. The gene expression analysis showed that Mel significantly modulated gene expression in the unstressed cells in the exponential growth phase, and also during various stress treatments.

## Introduction

Melatonin (Mel, *N*-acetyl-5-methoxytryptamine) is an indole-amine that is considered ubiquitous in most living organisms. Mel antioxidant properties have been widely studied in higher organisms such as mammals or plants. However, its antioxidant capacity does not seem to be limited to its ability to scavenge free radicals. It has been also reported that its presence is capable of stimulating the synthesis of other important intracellular antioxidants, such as glutathione (Rodriguez et al., 2004), inducing antioxidant enzymes by suppressing pro-oxidant enzymes, and improving the mitochondrial function and thereby reducing free radical formation (Acuña Castroviejo et al., 2011; Zhang and Zhang, 2014; Paradies et al., 2015). Gene expression modulation by Mel may underlie these functions, as described for mammalian copper zinc superoxide dismutase (CuZnSOD) and glutathione peroxidase (GPx), whose gene expression is modulated by Mel in a dose-dependent manner (Mayo et al., 2002).

Mel has been significantly detected in many food plants, so consequently it can now be considered a dietary component, even if its daily intake is very difficult to estimate (Iriti and Varoni, 2015). In the last few years its presence has been reported in fermented drinks, such as wine or beer, as a result of Mel content in the different vegetal sources used, but also notoriously as a result of yeast metabolism (Rodriguez-Naranjo et al., 2011, 2012). However, there is still uncertainty as to the conditions that trigger Mel production, and its biosynthetic pathway still remains unknown. For an organism like yeast, unraveling Mel production conditions and its effects is an exciting topic in the food science field because presence of Mel in food due to yeast activity can confer numerous health benefits (Iriti and Varoni, 2015). Moreover, the use of Mel-producing yeasts can provide other technological gains, such as application to post-harvest treatments as an antifungal control agent (Iriti and Varoni, 2014; Sun et al., 2015; Li et al., 2016; Ma et al., 2016; Xu et al., 2016).

*Saccharomyces cerevisiae* is the main yeast used in the winemaking process, where it is exposed to a number of stressors, each with the potential to cause cellular damage and impair fermentation performance (Gibson et al., 2007). One of the main stressors described during wine fermentation is oxidative stress (Auesukaree, 2017) and, the synthesis of Mel can be a response to cope with this stress, similarly to the role ascribed to other antioxidant molecules, e.g., glutathione, or a signaling molecule to trigger the molecular machinery of antioxidant response. Previous studies on the origins of Mel indicate that its primary function is strongly related to defense against oxidative stress (Tan et al., 2014). This antioxidant function is highly conserved among the organisms that produce this compound, and antioxidant protection is indicated as a possible function in aerobic non-vertebrates (Hardeland and Poeggeler, 2003). However, very little evidence is available that shows an advantageous effect of endogenous Mel at physiological concentrations in yeasts, especially in the first stages of growth. Above physiological concentrations, Mel can even produce moderate growth inhibition, which is presumably dose-dependent as demonstrated before for mammal cells (Koziol et al., 2005; Owsiak et al., 2010; Zhang and Zhang, 2014). Since the ideal conditions for endogenous Mel production remain unknown, we set up a Mel-charging method to emulate endogenous production and to perform our different experiments with Mel-charged cells after removing extracellular Mel. Before the oxidative stress treatments, we tested the growth performance of Mel-charged cells to assess growth improvement and we measured intracellular reactive oxygen species (ROS) levels by flow cytometry. As mentioned above, the presence of Mel can modulate the synthesis of many other molecules and enzymes that play an antioxidant role. This general triggering of antioxidant response can rely on transcriptional regulation. Thus we selected a group of four genes that encode the enzymes directly related to antioxidant activity in the cytoplasm (*SOD1, TRX2, GPX2*, and *CTT1*) and their four representatives in mitochondria (*SOD2, TRX3, GPX3*, and *CTA1*) to perform gene expression analyses under H_2_O_2_ oxidative stress for both Mel-treated and control cells.

Oxidative stress is closely related to UV radiation since radiation is a significant source of ROS and DNA damage (Farrugia and Balzan, 2012). In plants, Mel has been reported on numerous species. The largest amounts of Mel have been determined in oily seeds and highly UV-exposed plant organs, which suggests that Mel also serves as a UV protector and promotes seed viability (Hardeland, 2016). Thus we aimed to test the effect of Mel on UV-irradiated yeast as an interesting issue within the scope of Mel’s protective ability. Similarly to oxidative stress, we also performed expression analyses with two radiation-sensitive genes involved in DNA damage repair, namely *RAD18* and *RAD52*, using UVC light as a stress-triggering agent.

Recently, Vazquez et al. (2017) reported that Mel supplementation to the culture medium alleviates the oxidative stress generated in the stationary phase and up-regulates the gene expression of the antioxidant defense-related genes in *S. cerevisiae*. In our study, in order to clearly assess the protective role of intracellular Mel, we developed an intracellular Mel-charging method. These Mel-charged cells were then tested against oxidative and UV stress. Following this approach, we were able to prove that Mel is uptaken by yeast cells and these Mel-enriched cells improve its growth and tolerance to oxidative and UV stress by modulating the gene expression in both unstressed cells and during stress treatments. These results corroborate some of those previously reported by Vazquez et al. (2017) and provide new evidence for the protective role of this molecule against specific stresses in *S. cerevisiae*.

## Materials and Methods

### Yeast Strain and Growth Conditions

In this study, we used the *S. cerevisiae* BY4743 strain from EUROSCARF (Germany). All the precultures were grown in YPD medium (10 g L^-1^ yeast extract, 20 g L^-1^ peptone, 20 g L^-1^ glucose) overnight, and were then inoculated in synthetic complete (SC) medium to an OD_600_ = 0.1–0.2 and grown at 30°C with 300 rpm orbital shaking to the mid-exponential phase (OD_600_ = 0.5–1.0) before any treatment. SC was composed of 1.7 g L^-1^ yeast nitrogen base without ammonium sulfate and amino acids, 5 g L^-1^ ammonium sulfate, 20 g L^-1^ glucose and SC drop-out (Formedium, United Kingdom). The Mel 0.1 M stock solution in absolute ethanol was freshly prepared before each use. For growth monitoring purposes, 96-well plates were inoculated with the cells from different conditions to reach an initial OD_600_ = 0.1 (inoculum level of 1⋅10^6^ cells mL^-1^) in 0.25 mL of fresh SC medium, incubated at 28°C. The non-inoculated wells for each experimental series were also included on the microplate to determine, and to consequently subtract, the noise signal. OD_600_ was automatically recorded by a SPECTROStar^®^ (BMG Labtech, Germany) reader every 30 min. Growth parameters were calculated from each condition by directly fitting the OD measurements *versus* time to the reparameterized Gompertz equation proposed by Zwietering et al. (1990):

$$y=D\times e^{-e\left(\frac{\;\mu_{\max}\times e}{D}\right)\times (\lambda -t)+1}$$

where *y* = ln(OD*_t_*/OD_0_), OD_0_ is the initial OD, OD*_t_* is the OD at time *t, D* = ln(OD*_t_*/OD_0_) is the asymptotic maximum, μ_max_ is the maximum specific growth rate (h^-1^), and λ is the lag phase period (h). Generation time (GT) was calculated using the GT = ln(2)/μ_max_ equation. For the viability assays, cultures were diluted and plated on solid YPD media, while cell viability was determined by counting the total colony-forming units in the different treatments. All the experiments were carried out in triplicate.

### Treatment for Melatonin Uptake

Mel solution was added to the exponential-growing cultures in SC at three different concentrations (0.05, 0.1, and 20 mM) to establish a suitable dose for further assays. After 30 min, cells were pelleted and washed twice with sterile water to remove extracellular remains. The control cells were mock-treated with the same volume of ethanol as the Mel-treated ones. Cells were resuspended in a cold 50% (v/v) ethanol–water solution and ruptured by glass bead beating by applying three shaking cycles at 30 s^-1^ for 30 s in a Tehtnica MillMix 20 homogenizer (Tehtnica, Slovenia) at 4°C. Lysed cells were centrifuged at 5,000 × *g* and 4°C for 10 min to remove the insoluble particles. The supernatant was transferred to a new tube and stored at -20°C until analyzed.

### Melatonin Detection and Quantitation

Intracellular Mel was detected and quantified by HPLC-MS/MS as previously described (Muñiz-Calvo et al., 2016). An Acquity ultra-high performance liquid chromatography (U-HPLC; Waters, United States) was used with an Acquity UPLC BEH C18 (2.1 × 50 mm, 1.7 μm) column (Waters, United States) and mobile phases A (0.5% formic acid in water) and B (acetonitrile). The flow rate was 0.4 mL min^-1^ and the injection volume was 5 μL. The gradient program was as follows: 0–0.5 min, 95:5% (v/v), 0.5–3.5 min 0:100% (v/v), and 3.5–7 min 95:5% (v/v). The column temperature was set at 30°C. An ACQUITY^®^ TQD triple quadrupole mass spectrometer equipped with a Z-spray electrospray ionization source was used for detection purposes. Spectra were acquired in the positive ionization mode by the multiple-reaction monitoring method employing an interchannel delay of 0.07 s. The multiple-reaction method transitions for Mel were *m/z* 233 → 174.10 and *m/z* 233 → 216.10.

### Intracellular ROS Measurement by Flow Cytometry

After the above treatment for Mel uptake, we measured the intracellular ROS levels of the exponentially growing cells as described in Ballester-Tomás et al. (2015). Briefly, cells after 30 min treatment with Mel and the untreated controls were pelleted and resuspended (OD_600_ = 0.25) in sterile phosphate-buffered saline (PBS). Then dihydrorhodamine 123 (DHR 123, Sigma) was added at 5 μg mL^-1^ of cell culture from a 2.5 mg mL^-1^ stock solution in ethanol. Cells were incubated in the dark for 90 min at 28°C. Finally, cells were harvested, washed, resuspended in PBS and analyzed using the “Annexin V and Cell Death” channel of a flow cytometer Muse Cell Analyzer (Millipore, United States). The settings were adjusted using negative (DHR 123-untreated cells) and positive (4 mM H_2_O_2_/60 min-stressed cells treated with DHR 123) controls. Data are expressed as the percentage of cells that show DHR 123-positive staining. The data represent the mean ± SD of three different experiments.

### Yeast Growth and Viability After Stress Exposure

For the growth and viability assays with oxidative stress, both the control and Mel-treated cells were resuspended in PBS and incubated for 1 h (30°C, 300 rpm) with H_2_O_2_ to a final concentration of 10 mM. For UV stress, 2–3 × 10^8^ cells were resuspended in sterile PBS and dispersed on 90-mm Petri dishes at a depth of no more than 1 mm. Cells were irradiated with 106.5 and 248.5 J/m^2^ UVC (254 nm) under a Vilber VL-6.C filtered lamp (Fisher Biotec, Australia) using the lower radiation dose for the growth assays in liquid media, while the higher dose was used for the viability assays. After stresses, cells were washed twice and plated on solid media to assess viability, and were inoculated on a 96-well plate to record cell growth, as described above.

### Transcriptional Response Analyses

Transcriptional analyses by real-time PCR (qPCR) were performed with the Mel-treated and untreated cells challenged with H_2_O_2_ and UVC light. For the oxidative stress response analyses, Mel was added to growing cultures as described above. After 30 min of Mel treatment, H_2_O_2_ was added to the same culture at a final concentration of 3 mM. The samples for RNA extraction were taken before addition of H_2_O_2_ and at 5, 20, and 35 min after their H_2_O_2_ addition. For the UVC response analyses, samples were taken before 71 J/m^2^ irradiation and 5, 45, and 65 min after it. The selected H_2_O_2_ and UVC doses were deliberately lower than those used for the viability assays to avoid higher cell mortality and to evaluate the transcriptional response of cells during stress.

Total RNA extraction and the relative mRNA levels were determined as previously described (Sanvisens et al., 2014). Briefly, 10–20 mL of the exponentially growing cells were pelleted, washed and snap-frozen at -80°C. Cell pellets were ruptured by a Tehtnica MillMix 20 homogenizer (Tehtnica, Slovenia) in 0.4 mL of LETS buffer [0.1 M LiCl, 0.01 M EDTA, pH 8.0, 0.01 M Tris–HCl, pH 7.4, and 0.2% (wt/vol) SDS], 0.4 mL of phenol (pH 4.5)-chloroform (5:1) and 0.3 mL of glass beads. Supernatants were extracted with phenol–chloroform (5:1) and chloroform–isoamyl alcohol (24:1). RNA was twice precipitated overnight at -20°C, firstly by adding 2.5 volumes of 96% ethanol and 0.1 volume of 5 M LiCl, and secondly by adding 2.5 volumes of 96% ethanol and 0.1 volume of 3 M sodium acetate. RNA was finally resuspended in RNase-free MilliQ water and the concentration was determined in a NanoDrop spectrophotometer (Thermo Scientific, United States). Then 2.5 g of total yeast RNA was treated for 15 min at 25°C with DNase I RNase-free (Roche, Switzerland) according to the manufacturer’s protocol. Maxima Reverse Transcriptase (Thermo Scientific, United States) was used to synthesize cDNA from the DNase I-treated RNA following the manufacturer’s recommendations.

Quantitative real-time PCR was performed in a Light Cycler 480 II (Roche) using the SYBR Premix Ex Taq kit (TaKaRa, Japan) for fluorescent labeling. For this purpose, 2.5 μL cDNA were added to each reaction at a final volume of 10 μL. The real-time PCRs were performed using 0.2 μM of the corresponding oligonucleotides under the following conditions: 95°C for 10 s, followed by 40 cycles of 10 s at 95°C and 15 s at 55°C. At the end of the amplification cycles, a melting-curve analysis was conducted to verify the specificity of the reaction. A standard curve was made with serial dilutions of the cDNA sample (2 × 10^-1^, 1 × 10^-1^, 2 × 10^-2^, 1 × 10^-2^, 2 × 10^-3^, 1 × 10^-3^). The primers used to determine the transcript levels are represented in **Table 1**. The data and error bars represent the averages and standard deviations of three independent biological samples.

**Table 1:** The primers used for qPCR, oriented 5′–3′.

Gene	Primer sequences
*ACT1*	F: TCTGAGGTTGCTGCTTTGGT
	R: CCGACGATAGATGGGAAGACA
*CTT1*	F: ATTACATACGCCGCTCCATAC
	R: CAGTGTCTGGTGTACCACTTT
*GPX2*	F: CTTCACGCCGCAGTATAAAGA
	R: CCTGCTTCCCGAACTGATTAC
*SOD1*	F: TGCTGGTCCTCACTTCAATC
	R: TTCGTCCGTCTTTACGTTACC
*TRX2*	F: TTCTTCCATGCCTACCCTAATC
	R: GCAATAGCTTGCTTGATAGCAG
*CTA1*	F: TCTGCGGGTCTGCTATGTTT
	R: TCGGCACTACCTTTATCACCAC
*GPX3*	F: GAACCTGGCTCTGATGAAGAA
	R: GGTCCTCATTGCCACCATTA
*SOD2*	F: TGGGAACACGCCTACTACTT
	R: TCTTTCCAGTTGACCACATTCC
*TRX3*	F: GATGATGCAACCACACTTAACG
	R: GCCGTCACTTCACACTCTT
*RAD18*	F: AGGTTCATCGGACAGTTCAG
	R: TCGGTTCCCTGGTCACTTT
*RAD52*	F: TTCCAGCGAGTGTGCTAAAA
	R: TACTTGATTCCCAGCCCCTTC

*Forward and reverse primers are named F and R, respectively.*

### Statistical Analysis

Data are expressed as the mean values ± SD of at least three different experiments. Values were compared by a Student’s *t*-test. The 0.05 probability level was chosen as the maximum point of statistical significance throughout.

## Results

### Enrichment in Intracellular Mel and Its Impact on Growth and Intracellular ROS

**FIGURE 1:**
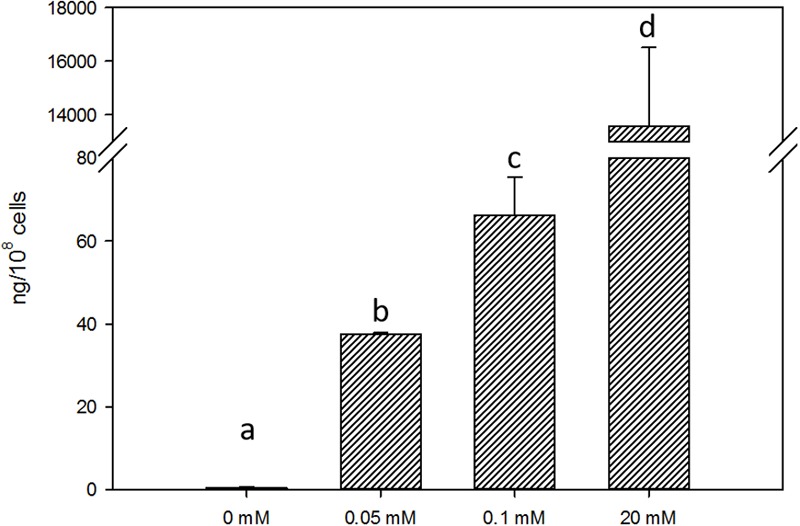
Yeast response to Mel treatment. Intracellular Mel detected in the control and Mel-treated cells with doses of 0.05, 0.1, and 20 mM. Values with a different letter are significantly different with a *P* < 0.05.

**FIGURE 2:**
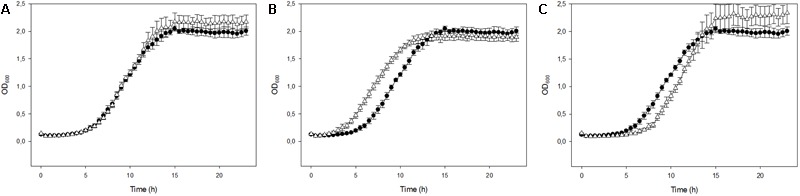
Cell growth for the control cells (circles) and Mel-treated cells (triangles) with concentrations of 0.05 **(A)**, 0.1 **(B)**, and 20 mM **(C)**.

**FIGURE 3:**
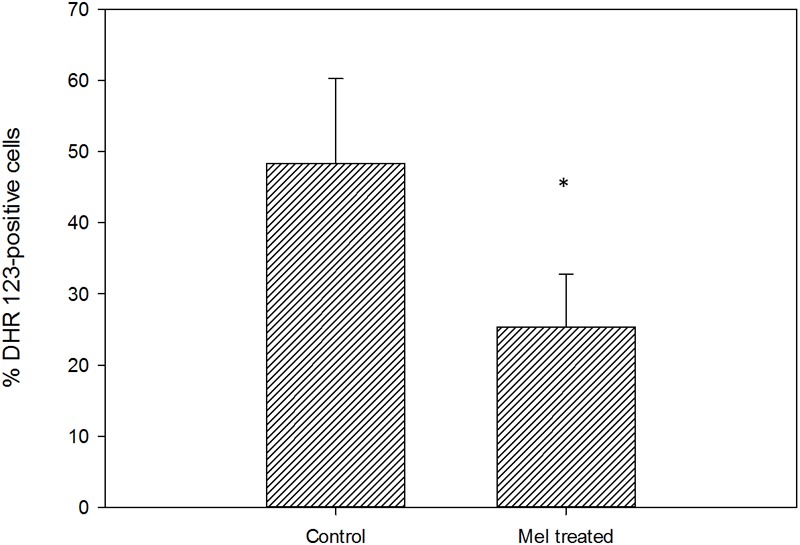
Intracellular ROS concentration in the control and Mel-treated cells with a dose of 0.1 mM, represented as the percentage of cells showing positive DHR 123 staining (^∗^*P* < 0.05).

The exponentially growing yeast cells were incubated in the presence of different Mel concentrations in order to enrich them with this molecule and to assess its impact on growth. The added Mel was uptaken by yeast cells, which showed a significant intracellular increase compared to the untreated control cells, with Mel levels of 37.60, 66.23, and 13,577.25 ng/10^8^ cells for the Mel-treated cells respectively for treatment doses 0.05, 0.1, and 20 mM (**Figure 1**). To assess whether enrichment in Mel can confer cells better fitness, the Mel-treated and untreated cells were grown in SC liquid media. These growth assays revealed a significant increase (*P* < 0.05) in the area under the curve (AUC) of the 0.1 mM Mel-treated cells *versus* the untreated cells (**Figure 2B**), while a decrease was observed for the 20 mM Mel-treated cells (**Figure 2C**) and no differences were found for the 0.05 mM treatment (**Figure 2A**). These differences in AUC were primarily due to changes in the lag phase of growth as there was no observable effect on the μ_max_ or the maximum OD obtained by the different cultures. As the 0.1 mM Mel-treated cells showed improved growth, we compared their intracellular ROS levels against the control cells and obtained a significant decrease of ROS in the Mel-charged cells during exponential growth (**Figure 3**).

### Protective Role of Mel Against Oxidative Stress and UV Radiation

**FIGURE 4:**
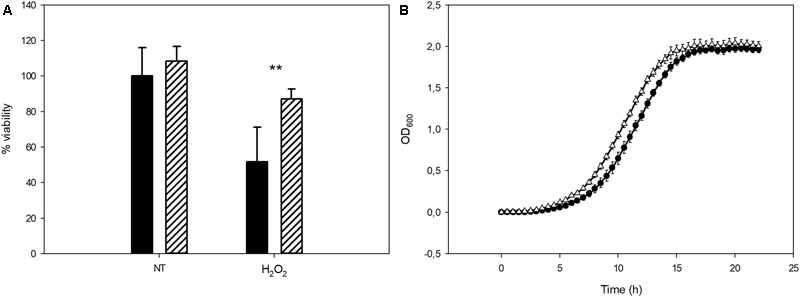
**(A)** Viability of yeast cells before (NT) and after stress (H_2_O_2_) for the control and Mel-treated cells (solid black and stripes, respectively). **(B)** Effect of H_2_O_2_ on the control (circles) and Mel-treated cells’ (triangles) growth. Asterisks denote significance for control compared to Mel-treated cells for each condition (^∗∗^*P* < 0.01).

To evaluate the possible role of Mel as an antioxidant agent in *S. cerevisiae*, the Mel-treated and untreated cells were subjected to oxidative stress shock by H_2_O_2_. After this oxidative agent was present for 1 h, cells were plated to determine the mortality percentage after oxidative stress shock. This oxidative stress resulted in the non-viability of approximately half the population in the control cells (not enriched in Mel). However, the intracellular presence of this molecule raised viability to 87%, which clearly indicates the amelioration of oxidative damage by this molecule being present (**Figure 4A**).

The same H_2_O_2_-challenged cells were also grown in SC to determine the impact of this stressor on yeast growth. Growth curves were used to determine the AUC of both the Mel-treated and untreated cells (**Figure 4B**). The Mel-treated cells had a significantly higher AUC than the control cells. Similarly to the above-mentioned growth of the unstressed cells, the main difference between both growth curves relied on a shorter lag phase in the Mel-treated than in the untreated ones.

**FIGURE 5:**
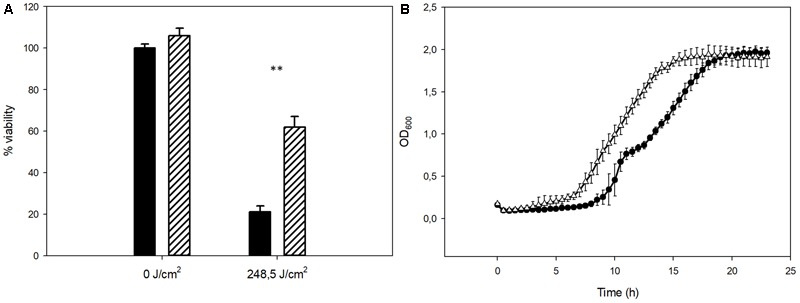
**(A)** Viability of yeast cells before (NT) and after UV radiation (248.5 J/cm^2^) for the control and Mel-treated cells (solid black and stripes, respectively). **(B)** Effect of UV radiation on the control (circles) and Mel-treated cells’ (triangles) growth. Asterisks denote significance for control compared to Mel-treated cells for each condition (^∗∗^*P* < 0.01).

As far as we know, Mel has not been connected with UV protection for yeast. To explore this possibility, the Mel-treated and untreated cells were irradiated with UVC light. According to the literature (Birrell et al., 2001), the dose used for viability assays provokes loss of viability above 50%. The viability and AUC of the UV-stressed cells were also determined as they were in oxidative stress shock. The viability of the untreated cells lowered to 21% when challenged with UVC light (248.5 J/m^2^), while the viability of the Mel-treated cells was significantly higher (62%) (**Figure 5A**). The growth curve of the UV-stressed cells also showed a higher AUC for the Mel-treated cells compared to the stressed control (**Figure 5B**).

### Transcriptional Response to Oxidative Stress and UV Radiation in the Mel-Enriched Cells

**FIGURE 6:**
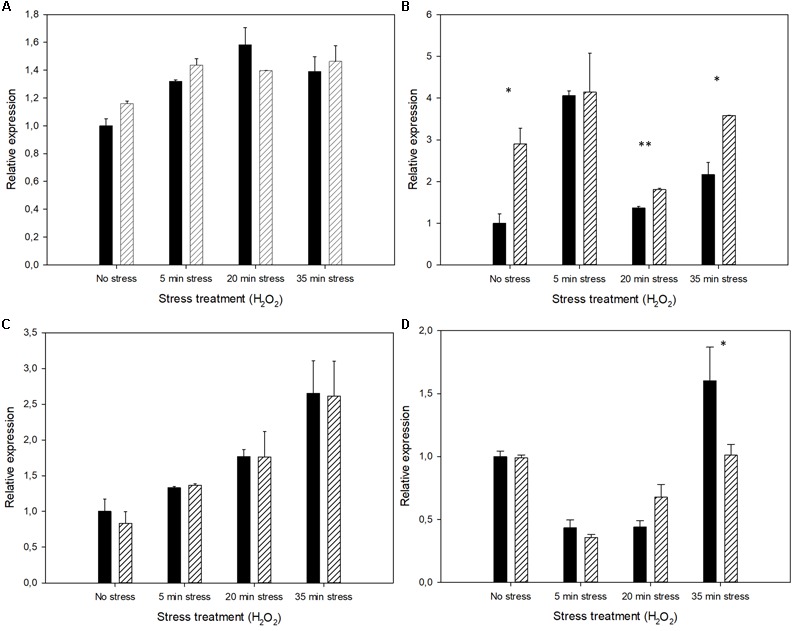
Expression profiles of the untreated cells (control) compared to the Mel-treated cells (solid black and stripes, respectively) showing the relative amount of the transcripts of the antioxidant genes with cytosolic activity **(A)** *SOD1*, **(B)** *TRX2*, **(C)** *GPX2*, and **(D)** *CTT1* under oxidative stress conditions (H_2_O_2_). Changes in gene activity are shown in relation to the expression of the untreated cells before stress exposure (set as value 1). Asterisks denote significance for control compared to Mel-treated cells for each time-point (^∗^*P* < 0.05; ^∗∗^*P* < 0.01).

**FIGURE 7:**
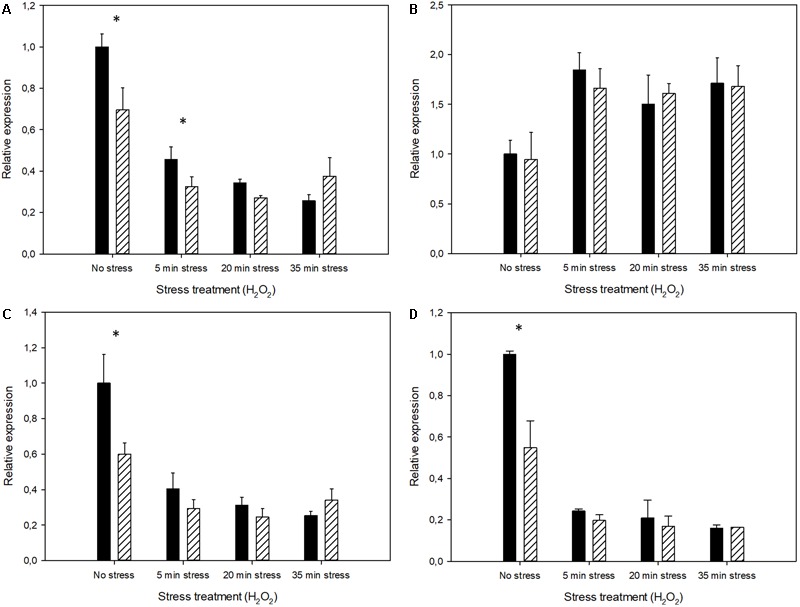
Expression profiles of the untreated cells (control) in comparison to the Mel-treated cells (solid black and stripes, respectively) showing the relative amount of the transcripts of the antioxidant genes with mitochondrial activity **(A)** *SOD2*, **(B)** *TRX3*, **(C)** *GPX3*, and **(D)** *CTA1* under oxidative stress conditions (H_2_O_2_). Changes in gene activity are shown in relation to the expression of the untreated cells before stress exposure (set as value 1). Asterisks denote significance for control compared to Mel-treated cells for each time-point (^∗^*P* < 0.05).

Quantitative PCR was used for the transcriptional analysis of those genes involved in antioxidant defense. As Mel strongly affects mitochondrial activity (Zhang and Zhang, 2014; Reiter et al., 2016), we selected four cytosolic genes (*SOD1, TRX2, GPX2*, and *CTT1*) and their counterparts, mitochondrial genes (*SOD2, TRX3, GPX3*, and *CTA1*), to analyze how Mel modulated their early transcriptional response against stress (**Figures 6, 7**). The first remarkable result was that the uptake of Mel in the exponentially growing cells, before the stress treatment, had already modified transcriptional activity by up-regulating cytosolic gene *TRX2* and down-regulating mitochondrial genes *SOD2, GPX3*, and *CTA1*. Contrarily to what was expected, oxidative shock did not always result in the immediate induction of these antioxidant genes. Most mitochondrial genes showed down-regulation immediately after oxidative stress (only *TRX3* was up-regulated), whereas cytosolic genes *SOD1, TRX2*, and *GPX2* showed higher transcript levels after the H_2_O_2_ incubation (*CTT1* was also transiently down-regulated after stress, followed by an immediate increase in transcripts). The Mel-treated cells showed a similar regulation trend as the untreated cells, but this up- or down-regulation was more buffered, with less marked changes in the relative expression. This smaller impact of oxidative shock on the Mel-charged cells can be explained because, as mentioned above, these genes were already activated by Mel uptake prior to the stress treatment. Thus intracellular Mel seemed to somehow prepare the transcriptional machinery to provide a quick response to oxidative stress.

**FIGURE 8:**
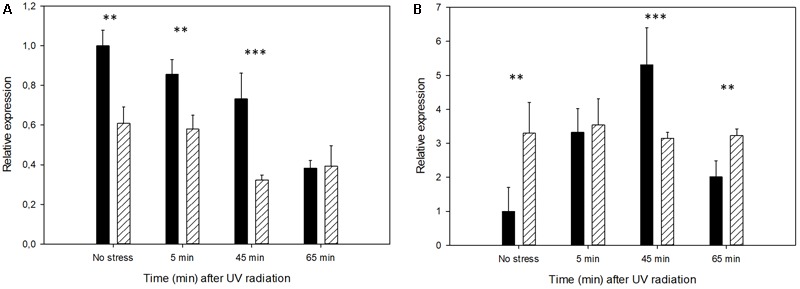
Expression profiles of the untreated cells (control) in comparison to the Mel-treated cells (solid black and stripes, respectively) showing the relative amount of the transcripts of the DNA-repairing genes **(A)** *RAD18* and **(B)** *RAD52* after UV irradiation. Changes in gene activity are shown in relation to the expression of the untreated cells before stress exposure (set as value 1). Asterisks denote significance for control compared to Mel-treated cells for each time-point (^∗∗^*P* < 0.01; ^∗∗∗^*P* < 0.001).

We also analyzed the gene expression levels of *RAD18* and *RAD52*, two sensitive genes to UV radiation that are involved in the repair of ionizing-radiation-induced DNA damage in *S. cerevisiae* (Bailly et al., 1997; Symington, 2002). As with the antioxidant genes, the Mel-treated cells showed differences in gene expression before being challenged with UV exposure (**Figure 8**), with the *RAD52* gene being threefold induced only by Mel uptake. UV exposure led to this gene being induced in the control cells, but minimal changes were observed in the Mel-treated cells. Conversely, *RAD18* was down-regulated after the UV exposure within the studied time frame. Thus similarly to antioxidant defense genes, the intracellular presence of Mel already modified the transcriptional activity by predisposing cells for UV stress.

## Discussion

Since Mel was initially discovered in a mammalian pineal gland, at that time it was believed to be exclusively produced in animals, particularly in the pineal gland of vertebrates. However, the availability of powerful analytical methods has detected the presence of Mel in numerous non-vertebrate taxa, including microorganisms like bacteria and fungi (Hardeland and Poeggeler, 2003; Tan et al., 2014). The recent connection made between the presence of Mel in wine and the metabolism of wine yeast opens up ways to improve the presence of this bioactive molecule, with multiple healthy properties for consumers in fermented beverages through the fermentative agent. However, it also poses a question mark: what is the physiological role of Mel synthesis in yeasts? Hardeland et al. (1995) have already excluded the main role described firstly in mammals as a regulator of the circadian cycle in an organism in which circadian behavior has not been clearly demonstrated, and may even be absent. Many studies have reported the potent antioxidant properties of Mel and relate its synthesis with defense against the oxidative stress produced during aerobic metabolism (Hardeland and Pandi-Perumal, 2005; Tan et al., 2014; Zhang and Zhang, 2014). Some reports have also connected the synthesis of Mel with UV light exposure in plants (Hardeland, 2016). To date however, there is not enough scientific evidence to clearly connect Mel synthesis in yeasts with antioxidant defense and/or UV damage. It was only until very recently that Vazquez et al. (2017) proved that Mel reduces oxidative stress damage to cells grown in the presence of Mel in the medium, and that it is able to up-regulate antioxidant genes in the stationary phase. In this study, we followed a different approach by using exponentially growing cells charged with Mel to assess the protective role of this molecule. Our results revealed how the presence of intracellular Mel improves growth and protects cells against oxidative and UV damage, which corroborates some of the results that Vazquez et al. (2017) obtained, but provides new evidences for the protective role of this molecule against different stresses. We also studied the immediate transcriptional response of the key genes involved in both cellular stresses.

When we started this study, we were unable to induce reproducible Mel synthesis in *S. cerevisiae*. Thus we decided to charge cells with Mel by incubating them during a short period of time (30 min) in the presence of this molecule. The analysis of the Mel-treated cells clearly showed an increase in intracellular Mel and, therefore, the capacity of yeast to take up this indole. The Mel intake mechanism in yeast is still unknown and there is no clear evidence as to whether this molecule can be uptaken by passive diffusion or by the active transport facilitated by any specific permease. Reiter and Tan (2003b) have reported that, given its amphiphilic nature, Mel can cross physiological barriers in both the lipid and aqueous environments of mammalian cells. Conversely, Hevia et al. (2015) have proved that members of the SLC2/GLUT family glucose transporters play a central role in Mel uptake in mice cells. Another question posed is whether the intracellular concentration determined in the Mel-treated cells is comparable to a physiological concentration of endogenous synthesis. Although a wide variation in the standard physiological concentration of intracellular Mel has been reported in yeasts, Reiter and Tan (2003a) consider that physiological levels of Mel can be acceptably variable, even when exceeding nanomolar ranges, which is consistent with the intracellular concentration detected in the Mel-treated cells we used for the stress assays (∼66 ng/10^8^ cells). In fact we are now able to induce endogenous Mel synthesis by pulses of tryptophan, and other intermediates of the route, and can obtain similar concentrations to that obtained in this study (data not shown; manuscript being prepared).

As we performed different growth assays with distinct intracellular Mel amounts, we chose the Mel dose that conferred yeast cells better growth performance for further assays. The first remarkable effect of the presence of intracellular Mel was the modulation of the lag phase, which depended on the treatment dose, showing a shortened lag phase for cells treated with a concentration of 0.1 mM and prior to stress treatments. It is difficult to consider explaining such advanced growth by using this molecule as a nutrient because its intracellular concentration is very low. Thus we may think of Mel as an inducer molecule that promotes growth. Mel as a trigger for an early start-up of cell growth poses interesting questions about its role as a signaling molecule involved in growth modulation in a population density-dependent manner. Although Mel has not been described as a quorum-sensing molecule in yeast, its presence can directly or indirectly modulate the yeast metabolism at different levels. Therefore, further insights into cell-to-cell communication and Mel membrane receptors are needed. In fact other molecules that derive from tryptophan metabolism, such as tryptophol and other aromatic alcohols, have been reported to act as quorum-sensing molecules (Chen and Fink, 2006; González et al., 2017).

As far as we know, this is the first report to show a significant protection of intracellular Mel in yeast against UV irradiation, with a marked reduction in cell mortality after exposure to radiation. Positive results about Mel’s protective effect against UV light have been recently reported for human melanocytes, which lends more support to this function of Mel (Janjetovic et al., 2017). Mel’s protective effect may occur due to several mechanisms. Mel can directly act as a direct scavenger to detoxify reactive oxygen (Reiter et al., 2001, 2016; Anisimov et al., 2006), but can indirectly reduce oxidative stress by increasing the activities of antioxidative defense systems by stimulating the synthesis of other important intracellular antioxidants, such as glutathione (Antolín et al., 1996; Rodriguez et al., 2004), by increasing the efficiency of the mitochondrial electron transport chain (Martín et al., 2000; León et al., 2005; López et al., 2009), and interacting synergistically with other antioxidants (Gitto et al., 2001; López-Burillo et al., 2003). Thus one point to look at in-depth in the near future is whether this protective role is the consequence of only direct antioxidant and photoprotectant properties, or if it is mainly a signaling molecule that triggers a molecular and physiological response to cope with these stress situations and to regulate cellular growth. The comparison of the transcriptional activity in Mel-treated and untreated cells directly indicates this signaling role.

The transcriptional activity of the genes involved in response to oxidative stress and UV damage was modified by intracellular Mel in the unstressed cells. When cells were subjected to the stress situation, the changes in gene activity were more buffered in the Mel-enriched cells, whereas more marked transcriptional changes took place in the non-enriched cells. These results seem to indicate that Mel prepares cells to better endure stress situations by early modifying the mRNA levels of these antioxidant and photoprotective genes. Vazquez et al. (2017) reported the induction of many antioxidant genes as an effect of the presence of Mel in the medium, and obtained higher transcript levels after 16 h of exposure to Mel. In our case, we studied the short transcriptional response of the key genes involved in oxidative and UV protection after cell exposure to these stresses. However, we observed no early induction of all the protective genes that we studied after stress exposure. We observed mainly a down-regulation of the genes that coded for mitochondrial enzymes, except for *TRX3*. This early down-regulation of the genes associated with mitochondrial function has also been observed as a transient effect in early stages of the stress response to other oxidants like cumene hydroperoxide, while other cytosolic antioxidant genes are up-regulated at the same time (Sha et al., 2013). Further studies into the gene expressions that cover a wider time range are likely to reveal the global up-regulation of redox and ROS-removing enzymes, as previously described (Gasch et al., 2000; Causton et al., 2001; Vazquez et al., 2017).

## Conclusion

In conclusion, this study supports the role of Mel as an antioxidant molecule in yeast, as previously described, and provides new evidences for its ability to confer yeast cells protection against oxidative stress. It describes for the first time in yeast the role of Mel in protection against UV radiation by lowering mortality and improving growth performance after stress. Among the possible mechanisms of action of Mel, we proved its ability to modify the gene expression of antioxidant and DNA-repairing genes under stress conditions. The transcriptional response of studied genes revealed that Mel itself provokes changes in expression, which seems to prepare cells against upcoming stress. Nevertheless, more insight into further gene expression profiles, plasma membrane transporters of Mel and endogenous synthesis conditions may shed some light on Mel’s biological importance, and reveal other features of this bioactive compound in yeast.

## Author Contributions

RB designed, performed, and analyzed the experiments, also discussed the results and wrote the manuscript. SM-C and JG designed the experiments, discussed the results, and wrote the manuscript.

## Conflict of Interest Statement

The authors declare that the research was conducted in the absence of any commercial or financial relationships that could be construed as a potential conflict of interest.
